# Supplementary material for: Protein allocation and utilization in the versatile chemolithoautotroph Cupriavidus necator
Source: eLife. 2021 Nov 1;10:e69019. doi: 10.7554/eLife.69019 (PMC8591527; doi:10.7554/eLife.69019)
Supplement: Supplementary file 1. [file elife-69019-supp1.docx]

**Supplementary file 1.** Table summarizing the constraints for the RBA model.

| **Parameter** | **Value** | **Unit** | **Reference** |
| --- | --- | --- | --- |
| Biomass composition | adapted from genome scale model | g gDCW^-1^ | Park et al., 2011 |
| replication efficiency (DNA pol III) | 2.88 × 10^6^ | nt/h | average of multiple sources, see Methods |
| transcription efficiency (RNA pol II) | 223,200 | nt/h | Epshtein et al., 2003 |
| ribosome efficiency | 97,200 | aa/h | Bulovic et al., 2019 |
| chaperone efficiency | 36045 × µ - 2888 | aa/h | Bulovic et al., 2019 |
| growth-associated maintenance | 150 | mmol gDCW^-1^ | Park et al., 2011 |
| non growth-associated maintenance | 3.0 | mmol gDCW^-1^ h^-1^ | Park et al., 2011 |
| total protein concentration  (in aa) | 6.18 | mmol gDCW^-1^ | Park et al., 2011 |
| fraction of cytoplasmic proteins | 0.8684 + 0.1060 × µ | unitless | this study |
| fraction of membrane proteins | 0.1316 - 0.1060 × µ | unitless | this study |
| fraction of non-enzymatic proteins in cytoplasm | 0.5374 - 0.5657 × µ | unitless | this study |
| fraction of non-enzymatic proteins in membrane | 0.8414 - 0.2147 × µ | unitless | this study |
| median k_app_ | 5770 | h^-1^ | this study |
