# Supplementary material for: Protein allocation and utilization in the versatile chemolithoautotroph Cupriavidus necator
Source: eLife. 2021 Nov 1;10:e69019. doi: 10.7554/eLife.69019 (PMC8591527; doi:10.7554/eLife.69019)
Supplement: Supplementary file 2. — Genes that were found to be essential in one or more conditions are marked with gray background. Fitness values below or above a threshold of |F| ≥ 3 are marked with red. Non-essential genes annotated for the same reactions were included for comparison. [file elife-69019-supp2.docx]

**Supplementary file 2.** Table of all annotated genes for the marked reactions in Figure 6B-D. Genes that were found to be essential in one or more conditions are marked with grey background. Fitness values below or above a threshold of |F| ≥ 3 are marked with red. Non-essential genes annotated for the same reactions were included for comparison.

| **Pathway** | **Reaction** | **Reaction name** | **Gene ID** | **Gene name** | **for-**  **mate** | **fruc-**  **tose** | **succi-**  **nate** |
| --- | --- | --- | --- | --- | --- | --- | --- |
| CBB cycle | FDH | Formate dehydrogenase | H16_A0640 | fdsG | -4.1 | -0.1 | -0.1 |
| CBB cycle | FDH | Formate dehydrogenase | H16_A0641 | fdsB | -4.4 | 0.1 | 0 |
| CBB cycle | FDH | Formate dehydrogenase | H16_A0642 | fdsA | -5.3 | -0.2 | -0.1 |
| CBB cycle | FDH | Formate dehydrogenase | H16_A0644 | fdsD | -6.3 | 0.3 | 0.1 |
| CBB cycle | FDH | Formate dehydrogenase | H16_A2934 | fdhC | -0.4 | 0.3 | 0.4 |
| CBB cycle | FDH | Formate dehydrogenase | H16_A2936 | fdhB1 | -0.1 | 0 | 0.4 |
| CBB cycle | FDH | Formate dehydrogenase | H16_A2937 | fdhA1 | -0.2 | 0.1 | 0.1 |
| CBB cycle | FDH | Formate dehydrogenase | H16_A3292 |  | -0.1 | -0.3 | -0.3 |
| CBB cycle | FDH | Formate dehydrogenase | H16_B1383 | cbbB | 0 | -0.3 | 0.3 |
| CBB cycle | FDH | Formate dehydrogenase | H16_B1452 | fdoG | -2.4 | 0.6 | 0.1 |
| CBB cycle | FDH | Formate dehydrogenase | H16_B1471 | fdhA2 | 0 | 0.1 | 0 |
| CBB cycle | FDH | Formate dehydrogenase | H16_B1700 | fdwA | -0.4 | 0.3 | -0.7 |
| CBB cycle | FDH | Formate dehydrogenase | H16_B1701 | fdwB | 0.2 | -0.6 | -0.7 |
| ED pathway | EDA | 2-dehydro-3-deoxy-phosphogluconate aldolase | H16_B1213 | eda | -2.6 | -4.3 | -1.7 |
| ED pathway | EDD | 6-phosphogluconate dehydratase | H16_A1178 | edd1 | -0.3 | -4.3 | -0.1 |
| ED pathway | EDD | 6-phosphogluconate dehydratase | H16_B2567 | edd2 | 0.1 | 0 | 0.2 |
| ED pathway | PGL | 6-phosphogluconolactonase | H16_B2565 | pgl | -2.2 | -3.9 | -1.1 |
| Pyruvate metabolism | ME1 | Malic enzyme (NAD) | H16_A3153 | maeA | 0 | 0.7 | -3.4 |
| Pyruvate metabolism | ME2 | Malic enzyme (NADP) | H16_A1002 | maeB | 0.3 | -0.2 | 0.2 |
| Pyruvate metabolism | PDH1 | Pyruvate dehydrogenase E1 component | H16_A1374 | pdhA | -2.6 | -5.2 | -3.2 |
| Pyruvate metabolism | PDH1 | Pyruvate dehydrogenase E1 component | H16_A1753 | pdhA2 | -0.4 | -1.1 | 0.1 |
| Pyruvate metabolism | PDH1 | Pyruvate dehydrogenase E1 component | H16_B0145 | acoB | -0.2 | -0.2 | 0.2 |
| Pyruvate metabolism | PDH1 | Pyruvate dehydrogenase E1 component | H16_B1300 | aceE | -0.1 | -0.3 | 0.4 |
| Pyruvate metabolism | PDH1 | Pyruvate dehydrogenase E1 component | H16_B2233 | bkdA1 | 0.2 | 0.2 | 0 |
| Pyruvate metabolism | PDH1 | Pyruvate dehydrogenase E1 component | H16_B2234 | bkdA2 | 0.2 | 0.3 | 0 |
| Pyruvate metabolism | PPC | Phosphoenolpyruvate carboxylase | H16_A2921 | ppc | -4.4 | -2.7 | -0.1 |
