# Supplementary material for: Protein allocation and utilization in the versatile chemolithoautotroph Cupriavidus necator
Source: eLife. 2021 Nov 1;10:e69019. doi: 10.7554/eLife.69019 (PMC8591527; doi:10.7554/eLife.69019)
Supplement: Supplementary file 3. [file elife-69019-supp3.docx]

**Supplementary file 3.** Table of the oligonucleotides/primers used in this study.

| Primer Name | Primer Sequence | Primer Function | Source |
| --- | --- | --- | --- |
| Short_Biotin_pHIMAR | (Biot)-CGCCCTGCAGGGATGTCCACGAG | Biotinylated forward primer for amplify Tn-specific sequences during TnSeq | This work |
| NC102 | GTGACTGGAGTTCAGACGTGTGCTCTTCCGATC | Illumina I7 specific reverse primer for use with Short_Biotin_pHIMAR for amplifying Tn-specific sequences during TnSeq | This work |
| Nspacer_barseq_pHIMAR | ATGATACGGCGACCACCGAGATCTACACTCTTTCCCTACACGACGCTCTTCCGATCTNNNNNNCGCCCTGCAGGGATGTCCACGAG | Tn specific primer containing Illumina adaptors I5 and P5 as primer extensions. | Wetmore et al., 2015 |
| NEBNext Index 3 Primer for Illumina | CAAGCAGAAGACGGCATACGAGATGCCTAAGTGACTGGAGTTCAGACGTGTGCTCTTCCGATC- | Illumina I7 specific reverse primer containing an index sequence and an I7 adaptor. | New England Biolabs |
| BarSeq_R_P2_UMI_Univ | AATGATACGGCGACCACCGAGATCTACACTCTTTCCCTACACGACGCTCTTCCGATCTNNNNNNGTCGACCTGCAGCGTACG | Barcode specific reverse phasing primer 1, used as a pool with the other phasing primers during BarSeq. Adds Illumina I5 and P5 adaptors. | This work |
| BarSeq_R_P2_UMI_Univ_N2 | AATGATACGGCGACCACCGAGATCTACACTCTTTCCCTACACGACGCTCTTCCGATCTNNGTCGACCTGCAGCGTACG | Barcode specific reverse phasing primer 2, used as a pool with the other phasing primers during BarSeq. Adds Illumina I5 and P5 adaptors. | This work |
| BarSeq_R_P2_UMI_Univ_N3 | AATGATACGGCGACCACCGAGATCTACACTCTTTCCCTACACGACGCTCTTCCGATCTNNNGTCGACCTGCAGCGTACG | Barcode specific reverse phasing primer 3, used as a pool with the other phasing primers using BarSeq. Adds Illumina I5 and P5 adaptors. | This work |
| BarSeq_R_P2_UMI_Univ_N4 | AATGATACGGCGACCACCGAGATCTACACTCTTTCCCTACACGACGCTCTTCCGATCTNNNNGTCGACCTGCAGCGTACG | Barcode specific reverse phasing primer 4, used as a pool with the other phasing primers during BarSeq. Adds Illumina I5 and P5 adaptors. | This work |
| BarSeq_R_P2_UMI_Univ_N5 | AATGATACGGCGACCACCGAGATCTACACTCTTTCCCTACACGACGCTCTTCCGATCTNNNNNGTCGACCTGCAGCGTACG | Barcode specific reverse phasing primer 5, used as a pool with the other phasing primers during BarSeq. Adds Illumina I5 and P5 adaptors. | This work |
| BarSeq_F_i7_001 | CAAGCAGAAGACGGCATACGAGATCGTGATGTGACTGGAGTTCAGACGTGTGCTCTTCCGATCTGATGTCCACGAGGTCTCT | Barcode specific forward indexing primer, used during BarSeq. Adds Illumina I7 and P7 adaptors. | This work |
| BarSeq_F_i7_002 | CAAGCAGAAGACGGCATACGAGATACATCGGTGACTGGAGTTCAGACGTGTGCTCTTCCGATCTGATGTCCACGAGGTCTCT | Barcode specific forward indexing primer, used during BarSeq. Adds Illumina I7 and P7 adaptors. | This work |
| BarSeq_F_i7_003 | CAAGCAGAAGACGGCATACGAGATGCCTAAGTGA CTGGAGTTCAGACGTGTGCTCTTCCGATCTGATGT CCACGAGGTCTCT | Barcode specific forward indexing primer, used during BarSeq. Adds Illumina I7 and P7 adaptors. | This work |
| BarSeq_F_i7_004 | CAAGCAGAAGACGGCATACGAGATTGGTCAGTGA CTGGAGTTCAGACGTGTGCTCTTCCGATCTGATGT CCACGAGGTCTCT | Barcode specific forward indexing primer, used during BarSeq. Adds Illumina I7 and P7 adaptors. | This work |
| BarSeq_F_i7_005 | CAAGCAGAAGACGGCATACGAGATCACTGTGTGA CTGGAGTTCAGACGTGTGCTCTTCCGATCTGATGT CCACGAGGTCTCT | Barcode specific forward indexing primer, used during BarSeq. Adds Illumina I7 and P7 adaptors. | This work |
| BarSeq_F_i7_006 | CAAGCAGAAGACGGCATACGAGATATTGGCGTGAC TGGAGTTCAGACGTGTGCTCTTCCGATCTGATGTC CACGAGGTCTCT | Barcode specific forward indexing primer, used during BarSeq. Adds Illumina I7 and P7 adaptors. | This work |
| BarSeq_F_i7_007 | CAAGCAGAAGACGGCATACGAGATGATCTGGTGA CTGGAGTTCAGACGTGTGCTCTTCCGATCTGATGT CCACGAGGTCTCT | Barcode specific forward indexing primer, used during BarSeq. Adds Illumina I7 and P7 adaptors. | This work |
| BarSeq_F_i7_008 | CAAGCAGAAGACGGCATACGAGATTCAAGTGTGAC TGGAGTTCAGACGTGTGCTCTTCCGATCTGATGTC CACGAGGTCTCT | Barcode specific forward indexing primer, used during BarSeq. Adds Illumina I7 and P7 adaptors. | This work |
| BarSeq_F_i7_009 | CAAGCAGAAGACGGCATACGAGATCTGATCGTGAC TGGAGTTCAGACGTGTGCTCTTCCGATCTGATGTC CACGAGGTCTCT | Barcode specific forward indexing primer, used during BarSeq. Adds Illumina I7 and P7 adaptors. | This work |
| BarSeq_F_i7_010 | CAAGCAGAAGACGGCATACGAGATAAGCTAGTGAC TGGAGTTCAGACGTGTGCTCTTCCGATCTGATGTC CACGAGGTCTCT | Barcode specific forward indexing primer, used during BarSeq. Adds Illumina I7 and P7 adaptors. | This work |
| BarSeq_F_i7_011 | CAAGCAGAAGACGGCATACGAGATGTAGCCGTGA CTGGAGTTCAGACGTGTGCTCTTCCGATCTGATGT CCACGAGGTCTCT | Barcode specific forward indexing primer, used during BarSeq. Adds Illumina I7 and P7 adaptors. | This work |
| BarSeq_F_i7_012 | CAAGCAGAAGACGGCATACGAGATTACAAGGTGAC TGGAGTTCAGACGTGTGCTCTTCCGATCTGATGTC CACGAGGTCTCT | Barcode specific forward indexing primer, used during BarSeq. Adds Illumina I7 and P7 adaptors. | This work |
| BarSeq_F_i7_013 | CAAGCAGAAGACGGCATACGAGATTTGACTGTGAC TGGAGTTCAGACGTGTGCTCTTCCGATCTGATGTC CACGAGGTCTCT | Barcode specific forward indexing primer, used during BarSeq. Adds Illumina I7 and P7 adaptors. | This work |
| BarSeq_F_i7_014 | CAAGCAGAAGACGGCATACGAGATGGAACTGTGA CTGGAGTTCAGACGTGTGCTCTTCCGATCTGATGT CCACGAGGTCTCT | Barcode specific forward indexing primer, used during BarSeq. Adds Illumina I7 and P7 adaptors. | This work |
| BarSeq_F_i7_015 | CAAGCAGAAGACGGCATACGAGATTGACATGTGAC TGGAGTTCAGACGTGTGCTCTTCCGATCTGATGTC CACGAGGTCTCT | Barcode specific forward indexing primer, used during BarSeq. Adds Illumina I7 and P7 adaptors. | This work |
| BarSeq_F_i7_016 | CAAGCAGAAGACGGCATACGAGATGGACGGGTGA CTGGAGTTCAGACGTGTGCTCTTCCGATCTGATGT CCACGAGGTCTCT | Barcode specific forward indexing primer, used during BarSeq. Adds Illumina I7 and P7 adaptors. | This work |
| BarSeq_F_i7_017 | CAAGCAGAAGACGGCATACGAGATCTCTACGTGAC TGGAGTTCAGACGTGTGCTCTTCCGATCTGATGTC CACGAGGTCTCT | Barcode specific forward indexing primer, used during BarSeq. Adds Illumina I7 and P7 adaptors. | This work |
| BarSeq_F_i7_018 | CAAGCAGAAGACGGCATACGAGATGCGGACGTGA CTGGAGTTCAGACGTGTGCTCTTCCGATCTGATGT CCACGAGGTCTCT | Barcode specific forward indexing primer, used during BarSeq. Adds Illumina I7 and P7 adaptors. | This work |
| BarSeq_F_i7_019 | CAAGCAGAAGACGGCATACGAGATTTTCACGTGAC TGGAGTTCAGACGTGTGCTCTTCCGATCTGATGTC CACGAGGTCTCT | Barcode specific forward indexing primer, used during BarSeq. Adds Illumina I7 and P7 adaptors. | This work |
| BarSeq_F_i7_020 | CAAGCAGAAGACGGCATACGAGATGGCCACGTGA CTGGAGTTCAGACGTGTGCTCTTCCGATCTGATGT CCACGAGGTCTCT | Barcode specific forward indexing primer, used during BarSeq. Adds Illumina I7 and P7 adaptors. | This work |
| BarSeq_F_i7_021 | CAAGCAGAAGACGGCATACGAGATCGAAACGTGA CTGGAGTTCAGACGTGTGCTCTTCCGATCTGATGT CCACGAGGTCTCT | Barcode specific forward indexing primer, used during BarSeq. Adds Illumina I7 and P7 adaptors. | This work |
| BarSeq_F_i7_022 | CAAGCAGAAGACGGCATACGAGATCGTACGGTGA CTGGAGTTCAGACGTGTGCTCTTCCGATCTGATGT CCACGAGGTCTCT | Barcode specific forward indexing primer, used during BarSeq. Adds Illumina I7 and P7 adaptors. | This work |
| BarSeq_F_i7_023 | CAAGCAGAAGACGGCATACGAGATCCACTCGTGA CTGGAGTTCAGACGTGTGCTCTTCCGATCTGATGT CCACGAGGTCTCT | Barcode specific forward indexing primer, used during BarSeq. Adds Illumina I7 and P7 adaptors. | This work |
| BarSeq_F_i7_024 | CAAGCAGAAGACGGCATACGAGATGCTACCGTGA CTGGAGTTCAGACGTGTGCTCTTCCGATCTGATGT CCACGAGGTCTCT | Barcode specific forward indexing primer, used during BarSeq. Adds Illumina I7 and P7 adaptors. | This work |
| BarSeq_F_i7_025 | CAAGCAGAAGACGGCATACGAGATATCAGTGTGAC TGGAGTTCAGACGTGTGCTCTTCCGATCTGATGTC CACGAGGTCTCT | Barcode specific forward indexing primer, used during BarSeq. Adds Illumina I7 and P7 adaptors. | This work |
| BarSeq_F_i7_026 | CAAGCAGAAGACGGCATACGAGATGCTCATGTGAC TGGAGTTCAGACGTGTGCTCTTCCGATCTGATGTC CACGAGGTCTCT | Barcode specific forward indexing primer, used during BarSeq. Adds Illumina I7 and P7 adaptors. | This work |
| BarSeq_F_i7_027 | CAAGCAGAAGACGGCATACGAGATAGGAATGTGAC TGGAGTTCAGACGTGTGCTCTTCCGATCTGATGTC CACGAGGTCTCT | Barcode specific forward indexing primer, used during BarSeq. Adds Illumina I7 and P7 adaptors. | This work |
| BarSeq_F_i7_028 | CAAGCAGAAGACGGCATACGAGATCTTTTGGTGAC TGGAGTTCAGACGTGTGCTCTTCCGATCTGATGTC CACGAGGTCTCT | Barcode specific forward indexing primer, used during BarSeq. Adds Illumina I7 and P7 adaptors. | This work |
| BarSeq_F_i7_029 | CAAGCAGAAGACGGCATACGAGATTAGTTGGTGAC TGGAGTTCAGACGTGTGCTCTTCCGATCTGATGTC CACGAGGTCTCT | Barcode specific forward indexing primer, used during BarSeq. Adds Illumina I7 and P7 adaptors. | This work |
| BarSeq_F_i7_030 | CAAGCAGAAGACGGCATACGAGATCCGGTGGTGA CTGGAGTTCAGACGTGTGCTCTTCCGATCTGATGT CCACGAGGTCTCT | Barcode specific forward indexing primer, used during BarSeq. Adds Illumina I7 and P7 adaptors. | This work |
| BarSeq_F_i7_031 | CAAGCAGAAGACGGCATACGAGATATCGTGGTGAC TGGAGTTCAGACGTGTGCTCTTCCGATCTGATGTC CACGAGGTCTCT | Barcode specific forward indexing primer, used during BarSeq. Adds Illumina I7 and P7 adaptors. | This work |
| BarSeq_F_i7_032 | CAAGCAGAAGACGGCATACGAGATTGAGTGGTGA CTGGAGTTCAGACGTGTGCTCTTCCGATCTGATGT CCACGAGGTCTCT | Barcode specific forward indexing primer, used during BarSeq. Adds Illumina I7 and P7 adaptors. | This work |
| BarSeq_F_i7_033 | CAAGCAGAAGACGGCATACGAGATCGCCTGGTGA CTGGAGTTCAGACGTGTGCTCTTCCGATCTGATGT CCACGAGGTCTCT | Barcode specific forward indexing primer, used during BarSeq. Adds Illumina I7 and P7 adaptors. | This work |
| BarSeq_F_i7_034 | CAAGCAGAAGACGGCATACGAGATGCCATGGTGA CTGGAGTTCAGACGTGTGCTCTTCCGATCTGATGT CCACGAGGTCTCT | Barcode specific forward indexing primer, used during BarSeq. Adds Illumina I7 and P7 adaptors. | This work |
| BarSeq_F_i7_035 | CAAGCAGAAGACGGCATACGAGATAAAATGGTGAC TGGAGTTCAGACGTGTGCTCTTCCGATCTGATGTC CACGAGGTCTCT | Barcode specific forward indexing primer, used during BarSeq. Adds Illumina I7 and P7 adaptors. | This work |
| BarSeq_F_i7_036 | CAAGCAGAAGACGGCATACGAGATTGTTGGGTGA CTGGAGTTCAGACGTGTGCTCTTCCGATCTGATGT CCACGAGGTCTCT | Barcode specific forward indexing primer, used during BarSeq. Adds Illumina I7 and P7 adaptors. | This work |
