# Supplementary material for: Protein allocation and utilization in the versatile chemolithoautotroph Cupriavidus necator
Source: eLife. 2021 Nov 1;10:e69019. doi: 10.7554/eLife.69019 (PMC8591527; doi:10.7554/eLife.69019)
Supplement: Transparent reporting form [file elife-69019-transrepform1.pdf]

Westbrook Centre
Milton Road
Cambridge CB4 1YG, UK

P 01223 855340
W elifesciences.org
T @elife

eLife's transparent reporting form

We encourage authors to provide detailed information *within their submission* to facilitate the interpretation and replication of experiments. Authors can upload supporting documentation to indicate the use of appropriate reporting guidelines for health-related research (see EQUATOR Network), life science research (see the BioSharing Information Resource), or the ARRIVE guidelines for reporting work involving animal research. Where applicable, authors should refer to any relevant reporting standards documents in this form.

If you have any questions, please consult our Journal Policies and/or contact us: editorial@elifesciences.org.

Sample-size estimation

- You should state whether an appropriate sample size was computed when the study was being designed
- You should state the statistical method of sample size computation and any required assumptions
- If no explicit power analysis was used, you should describe how you decided what sample (replicate) size (number) to use

Please outline where this information can be found within the submission (e.g., sections or figure legends), or explain why this information doesn't apply to your submission:

> Information about the sample size and its estimation can be found in the methods section, subsection “Statistical analysis”. No power analysis was performed.

Replicates

- You should report how often each experiment was performed
- You should include a definition of biological versus technical replication
- The data obtained should be provided and sufficient information should be provided to indicate the number of independent biological and/or technical replicates
- If you encountered any outliers, you should describe how these were handled
- Criteria for exclusion/inclusion of data should be clearly stated
- High-throughput sequence data should be uploaded before submission, with a private link for reviewers provided (these are available from both GEO and ArrayExpress)

> Reproduction of experiments with identical or similar conditions (e.g. for BarSeq) is mentioned in the Results section of the main text.
> The number of biological replicates and the stage of replication is described in the Methods section, subsection “Statistical analysis”. Additionally, the number of replicates is stated in all figure legends. No outliers were removed.
> All processed data and analysis pipelines are available through a dedicated github repository at https://github.com/m-jahn/R-notebook-ralstonia-proteome.
> All raw data have been uploaded to the repositories PRIDE (for protein mass spectrometry) and ENA (for NGS data). The accession numbers for data submissions are included in the Methods section, subsection “Data and software availability”.

Please outline where this information can be found within the submission (e.g., sections or figure legends), or explain why this information doesn't apply to your submission:

eLife Sciences Publications, Ltd is a limited liability non-profit non-stock corporation incorporated in the State of Delaware, USA, with company number 5030732, and is registered in the UK with company number FC030576 and branch number BR015634 at the address Westbrook Centre, Milton Road, Cambridge CB4 1YG.

Statistical reporting

- Statistical analysis methods should be described and justified
- Raw data should be presented in figures whenever informative to do so (typically when N per group is less than 10)
- For each experiment, you should identify the statistical tests used, exact values of N, definitions of center, methods of multiple test correction, and dispersion and precision measures (e.g., mean, median, SD, SEM, confidence intervals; and, for the major substantive results, a measure of effect size (e.g., Pearson's r, Cohen's d)
- Report exact p-values wherever possible alongside the summary statistics and 95% confidence intervals. These should be reported for all key questions and not only when the p-value is less than 0.05.

Please outline where this information can be found within the submission (e.g., sections or figure legends), or explain why this information doesn't apply to your submission:

General statistical methods are described in the Methods section, subsection "Statistical analysis". Statistical metrics for centrality and dispersion are also indicated in the figure legends.
The type of statistical test for significance is indicated in figure legends, along with the exact p-value.

(For large datasets, or papers with a very large number of statistical tests, you may upload a single table file with tests, Ns, etc., with reference to sections in the manuscript.)

Group allocation

- Indicate how samples were allocated into experimental groups (in the case of clinical studies, please specify allocation to treatment method); if randomization was used, please also state if restricted randomization was applied
- Indicate if masking was used during group allocation, data collection and/or data analysis

Please outline where this information can be found within the submission (e.g., sections or figure legends), or explain why this information doesn't apply to your submission:

Not applicable.

Additional data files ("source data")

- We encourage you to upload relevant additional data files, such as numerical data that are represented as a graph in a figure, or as a summary table
- Where provided, these should be in the most useful format, and they can be uploaded as "Source data" files linked to a main figure or table
- Include model definition files including the full list of parameters used
- Include code used for data analysis (e.g., R, MatLab)
- Avoid stating that data files are "available upon request"

Please indicate the figures or tables for which source data files have been provided:

No source files are provided. All raw data, output tables, individual data points for figures, and analysis pipelines are provided through a dedicated github repository at https://github.com/m-jahn/R-notebook-ralstonia-proteome.
The definition of the RBA model and all its parameters is available at https://github.com/m-jahn/Bacterial-RBA-models . Table S1 of the Supplementary Information lists the main parameters of the model. Table S2 of the Supplementary Information lists fitness values for all significantly depleted genes, presented in Figure 6.

eLife Sciences Publications, Ltd is a limited liability non-profit non-stock corporation incorporated in the State of Delaware, USA, with company number 5030732, and is registered in the UK with company number FC030576 and branch number BR015634 at the address Westbrook Centre, Milton Road, Cambridge CB4 1YG.
